# Pleomorphic rhabdomyosarcoma of the liver in an adult: a rare case report

**DOI:** 10.1186/s12893-020-00742-7

**Published:** 2020-04-21

**Authors:** Mitsuyoshi Okazaki, Hidehiro Tajima, Yoshinao Ohbatake, Hiroyuki Shinbashi, Shinichi Nakanuma, Isamu Makino, Itasu Ninomiya, Sachio Fushida, Koushiro Ohtsubo, Tetsuo Ohta

**Affiliations:** 1 Division of Cancer Medicine, Department of Gastroenterological Surgery, Graduate School of Medical Science, Kanazawa University, 13-1 Takara-machi, Kanazawa, Ishikawa 920-8641 Japan; 2 Division of Medical Oncology Cancer Research Institute, Kanazawa University, 13-1 Takara-machi, Kanazawa, Ishikawa 920-8641 Japan

**Keywords:** Pleomorphic rhabdomyosarcoma, Heterogeneous mass in liver, Right hepatic lobectomy, Case report

## Abstract

**Background:**

Rhabdomyosarcoma (RMS), a malignant neoplasm that normally differentiates to form striated muscle, is the most common type of childhood soft tissue sarcoma. However, it infrequently occurs in adults and is uncommon in the liver. We herein report a case of RMS of the liver in an adult.

**Case presentation:**

A 73-year-old woman was admitted to our institution for investigation of a hepatic mass. She had been followed for primary biliary cirrhosis for the past 20 years. A contrast-enhanced computed tomography scan of the abdomen showed a 12- × 10-cm heterogeneous low-density mass lesion containing cystic and solid components. A percutaneous liver biopsy was performed, and poorly differentiated cancer containing an RMS cell-like component was observed. The patient was diagnosed with RMS of the liver, and open surgery with right hepatic lobectomy was performed. Histopathological examination confirmed a diagnosis of pleomorphic RMS of the liver. The patient died of rapid progression of the tumor 6 months after the operation.

**Conclusions:**

The tumor site in the present case is rare. The details of this case add to the current evidence base regarding establishment of the standard diagnosis and treatment of this rare condition. We recommend consideration of RMS as a differential diagnosis for hepatic tumors.

## Background

Rhabdomyosarcoma (RMS) is a malignant neoplasm that normally differentiates to form striated muscle. RMS is the most common type of childhood soft tissue sarcoma, constituting 5 to 10% of all solid tumors in childhood. However, it rarely occurs in adults; soft tissue sarcomas account for less than 1% of all cancers in adults [1–3]. Although this tumor may occur anywhere in the body, it is uncommon in the liver.

We herein report the clinicopathological features of a case of RMS of the liver in a 73-year-old woman.

## Case presentation

A 73-year-old woman presented with a fever and a 2-month history of right upper abdominal pain. The patient had been followed for primary biliary cirrhosis for the past 20 years and was being treated with ursodeoxycholic acid. A computed tomography (CT) scan performed by the previous doctor revealed a liver abscess, which was drained from the right hypochondriac region; however, the patient’s symptoms did not improve. She was admitted to our institution for further investigation of a hepatic mass. Physical examination revealed a right upper abdominal mass, but no anemia or jaundice.

Laboratory data showed an elevated C-reactive protein level (7.6 mg/dL). The hemoglobin concentration, white blood cell count, platelet count, electrolyte levels, liver enzyme levels, and bilirubin level were within the reference range. The serum levels of α-fetoprotein and PIVKA-II were 4 ng/mL and 43 U/mL, respectively.

**Fig. 1:**
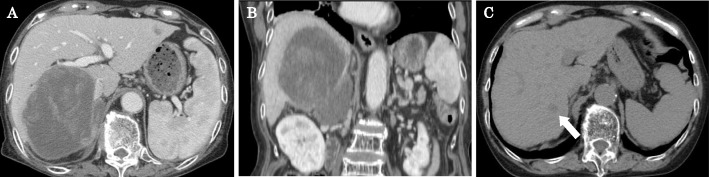
Abdominal contrast-enhanced computed tomography showing a heterogeneous mass lesion in the right lobe of the liver; **a** axial section, **b** coronal section. **c** Four months previously, a 2-cm low-density tumor (arrow) was detected in liver segment 6

An abdominal contrast-enhanced CT scan revealed a 12- × 10-cm heterogeneous low-density mass lesion containing cystic and solid components with post-contrast enhancement in the solid component (Fig. 1a, b). This mass occupied the right lobe of the liver, and a large component of the lesion was present in the right subhepatic space. We determined that the tumor originated in the liver because a CT scan performed for follow-up of the patient’s primary biliary cirrhosis 4 months previously had revealed a 2-cm low-density tumor in liver segment 6 (Fig. 1c). A percutaneous liver biopsy was performed, and poorly differentiated cancer containing an RMS cell-like component was diagnosed.

**Fig. 2:**
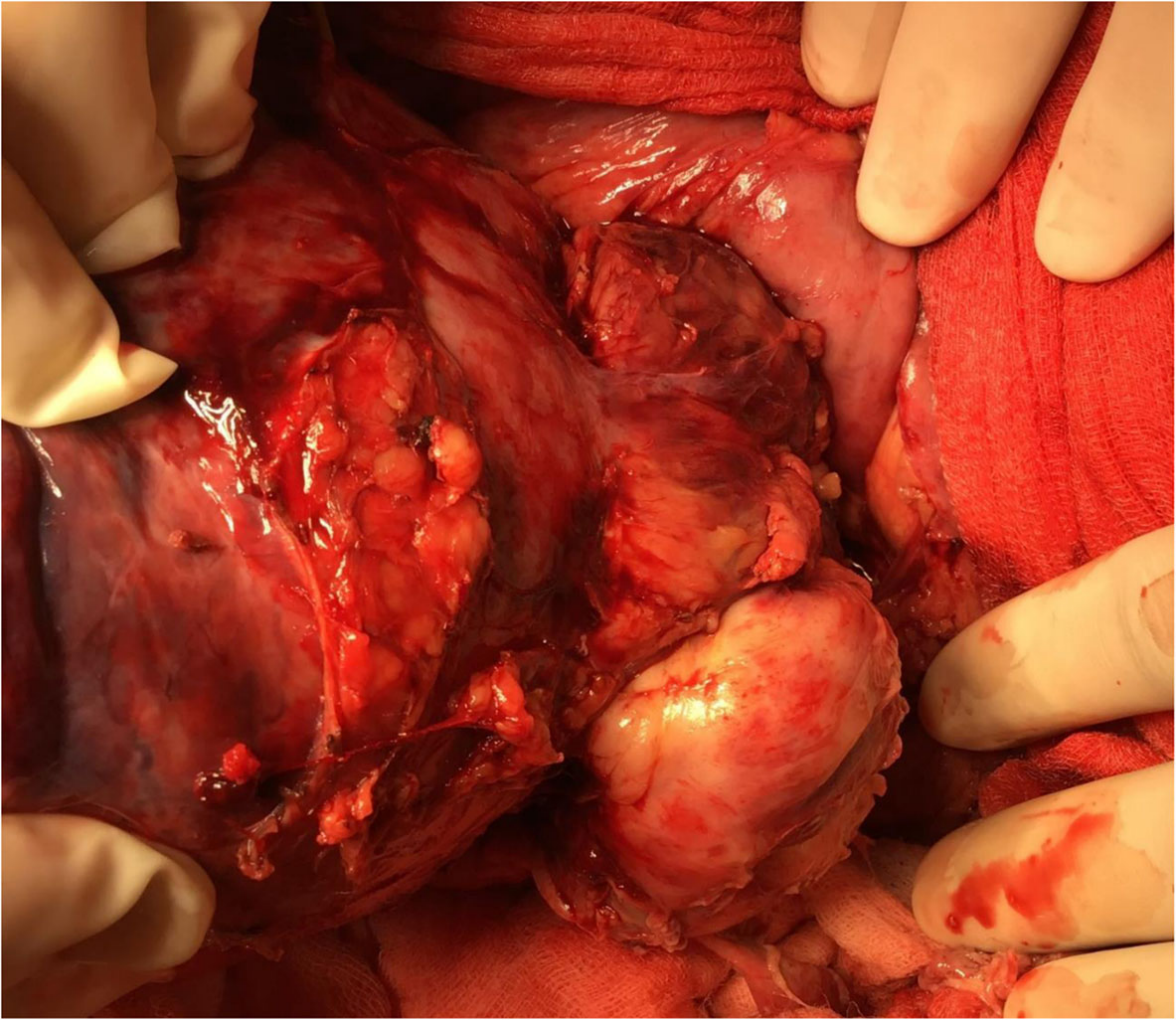
Intraoperative view of resected mass

**Fig. 3:**
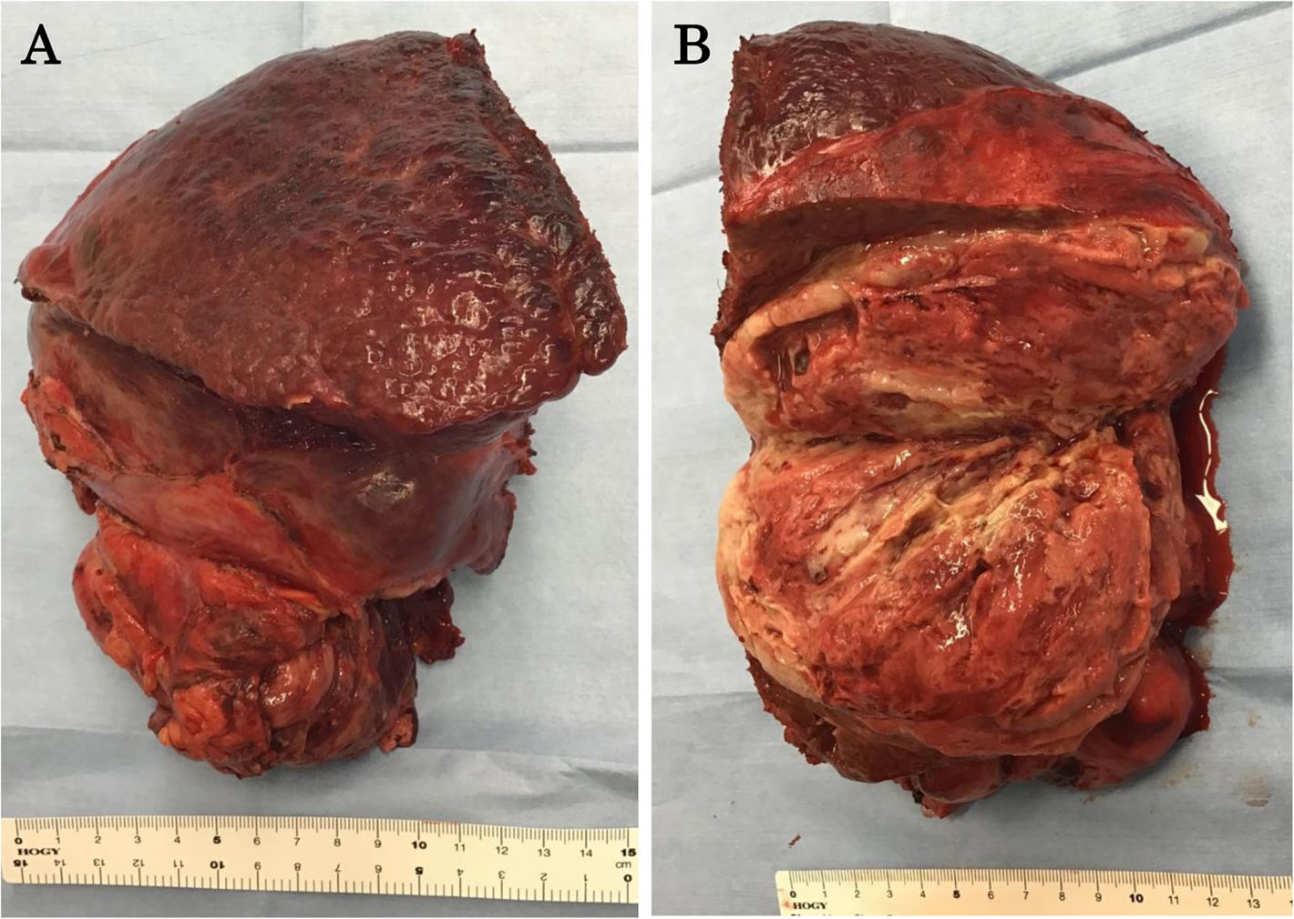
(**a**) Postoperative specimen of rhabdomyosarcoma of the liver. **b** Cut surface showed a tan-brown solid, friable tumor

**Fig. 4:**
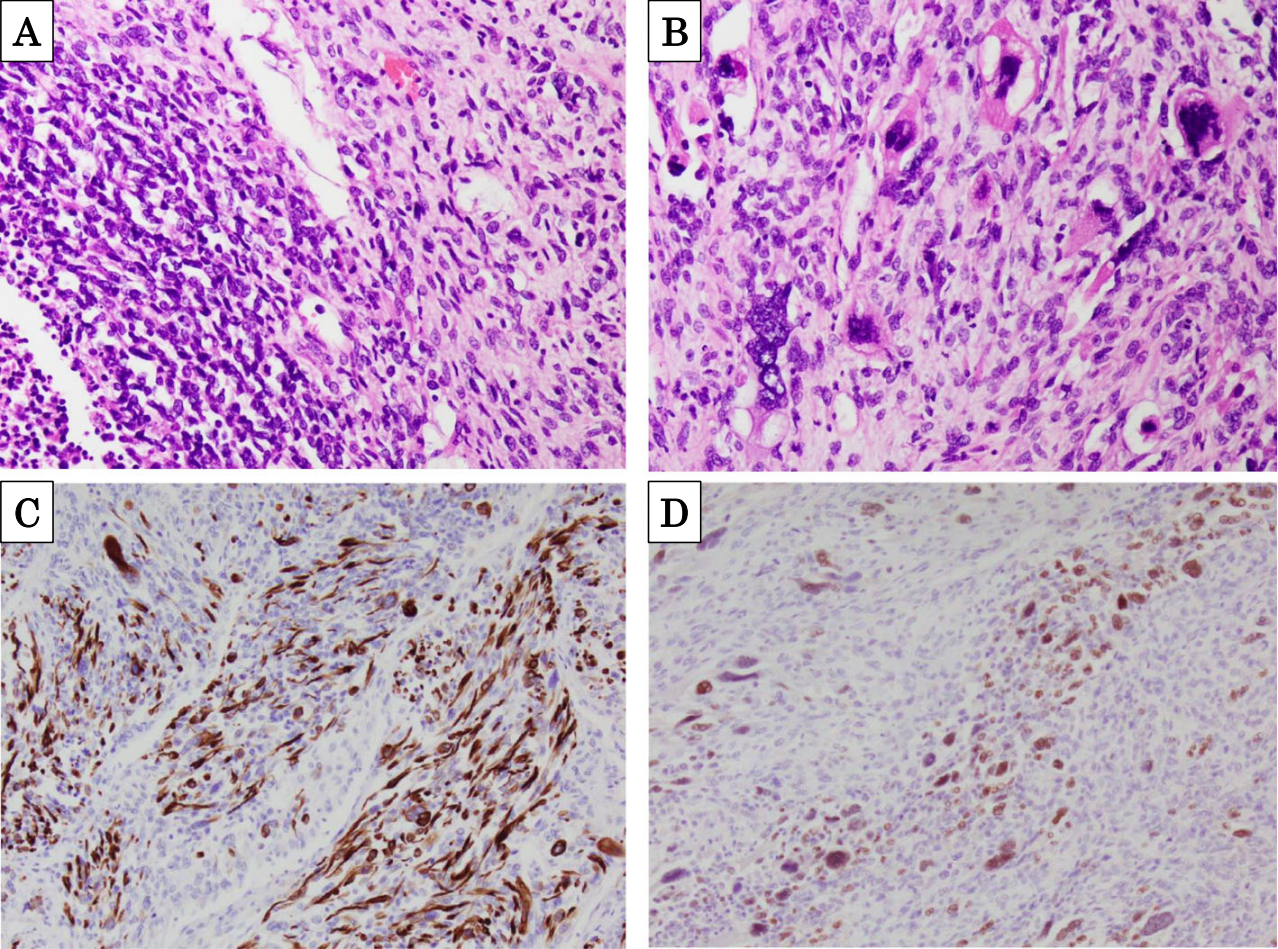
(**a**, **b**) Histopathological examination showed pleomorphic nuclei and spindle cells. Immunohistochemical analysis revealed (**c**) desmin positivity and (**d**) myogenin positivity

The patient underwent open surgery with right hepatic lobectomy. The intraoperative findings confirmed a tumor occupying the right lobe of the liver and no infiltration of the surrounding organs (Fig. 2). Examination of the gross specimen revealed a multilobulated tumor with a solid component (Fig. 3a, b). Histopathological examination of the tissue showed haphazardly oriented, large and small irregularities and pleomorphic or round cells containing abundant and eccentric eosinophilic cytoplasm and small oval nuclei with a prominent nucleolus. Immunohistochemical analysis showed desmin, myogenin, and myoglobin positivity and cytokeratin negativity (Fig. 4a–d). Based on these findings, the patient was diagnosed with pleomorphic RMS of the liver.

**Fig. 5:**
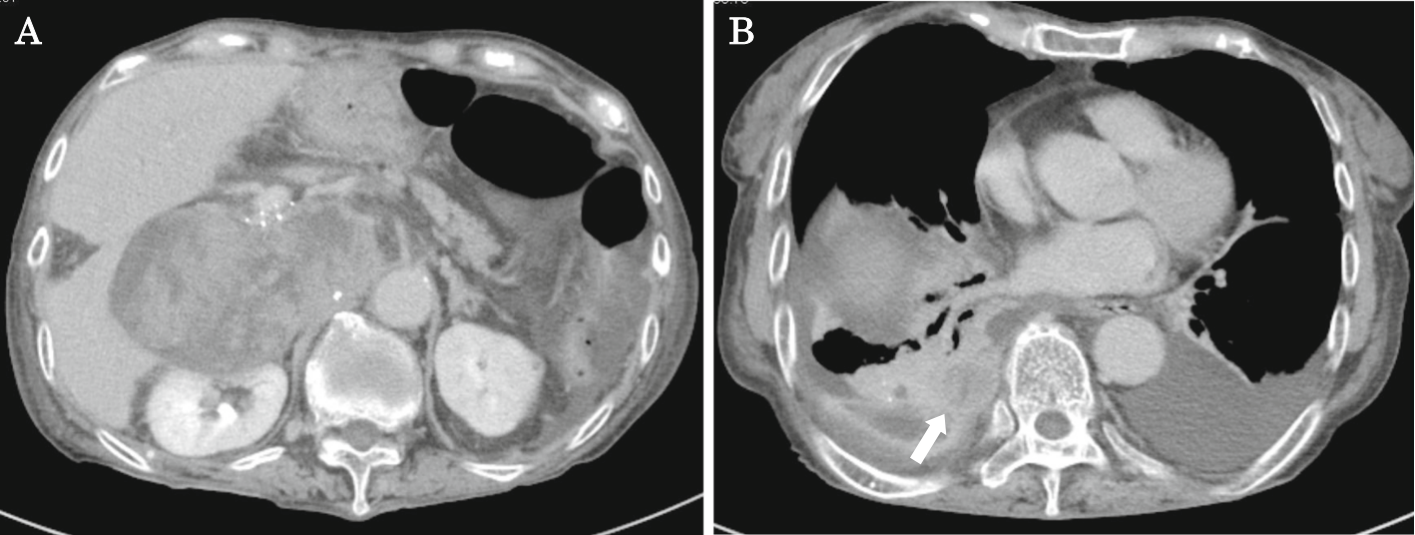
Computed tomography showed 8-cm low-density tumor compressing the inferior vena cava in the (**a**) liver resection area and (**b**) peritoneal space (arrow)

No complications occurred in the postoperative period, and the patient was discharged on the 28th postoperative day. Two months after the operation, an abdominal CT scan showed an 8-cm low-density tumor in the liver resection area compressing the inferior vena cava and peritoneal dissemination in the drainage route for diagnosis of the liver abscess before admission to our institution (Fig. 5a, b). The patient received one course of 70% dose trabectedin. Despite an initial good response to chemotherapy, she complained of severe adverse effects including loss of appetite and fatigue, and she rejected further chemotherapy. She subsequently experienced rapid progression of the tumor and died of malnutrition and multiple organ failure 6 months after the operation.

## Discussion and conclusions

RMS in the liver, especially that in adults, is difficult to manage because of the absence of standard diagnostic criteria or a standard treatment protocol. Only 10 cases of RMS of the liver in adults, including our case, have been reported to date and are summarized in Table 1 [4–12]. Among these cases, RMS was more common in men than women, and our case involved the oldest patient.

**Table 1:** Reported cases of rhabdomyosarcoma of the liver in adults

Authors	Year	Age, years	Sex	Extent of liver involvement	Histology	Treatment	Outcome
Miller and Pack [4]	1956					Total right hepatic lobectomy	
Goldman and Freiedman [5]	1969	65	Male	Autopsy: right lobe contained an ovoid tumor measuring 35 × 15 × 10 cm	Embryonal/alveolar	Symptomatic treatment without any surgical procedure or chemotherapy	Died 3 months from initial symptoms
Watanabe et al. [6]	1983	70	Male	Autopsy: yellowish-brown multinodular tumors up to 5 cm in diameter in right lobe	Pleomorphic	Symptomatic treatment without any surgical procedure or chemotherapy	Died 8 months from initial symptoms
McArdle et al. [7]	1989	53	Male	Large mass occupied the entire right lobe	Embryonal	Surgical resection	Died 3 months from initial symptoms
Akasofu et al. [8]	1999	52	Male	Autopsy: 19- × 12- × 11-cm tumor occupied almost the entire right lobe; it was not encapsulated and had invaded the right adrenal gland, diaphragm, bilateral hepatic ducts, and inferior vena cava		Symptomatic treatment without any surgical procedure or chemotherapy	Died 2.5 months from initial symptoms
Schoofs et al. [9]	2011	59	Female		Alveolar	Primary surgical resection + chemotherapy (doxorubicin/ifosfamide)	Initial good response to chemotherapy and stable disease at 12 months after diagnosis; died 31 months after the first symptoms
Aassab et al. [10]	2012	25	Male	Lesion in the right lobe of the liver measuring 136 mm	Embryonal	Biopsy followed by chemotherapy: doxorubicin, ifosfamide, and vincristine	Died after 3 months from initial symptoms
Arora et al. [11]	2016	67	Male	14.5- × 12.3- × 9.1-cm lesion involving left hepatic lobe. Large component of lesion was seen bulging into the left subhepatic space	Embryonal	Left hepatic lobectomy followed by adjuvant chemotherapy: doxorubicin, ifosfamide, and vincristine	At 24 months of follow-up, patients free from local recurrence and distant metastasis.
Yin et al. [12]	2018	66	Female	Large mass measuring about 20 × 15 cm in the right lobe of the liver	Pleomorphic	Emergency laparotomy for hemostasis and right hepatic lobectomy without adjuvant chemotherapy	Died 3 months from surgery
Present case	2019	73	Female	12- × 10-cm lesion involving right hepatic lobe	Pleomorphic	Primary surgical resection + chemotherapy (trabectedin)	Died 6 months from surgery

Horn and Enterline et al. [13] reported four subgroups of RMS: embryonal, alveolar, pleomorphic, and botryoid. Botryoid RMS is actually a subtype of embryonal RMS [14]. Embryonal RMS is the most frequent type of RMS in young children, alveolar RMS is the most frequent type in patients older than 10 years, and pleomorphic RMS is the most frequent type in advanced-age adults [3, 13]. Among the adult patients in whom RMS originated in the liver, four had embryonal RMS and three had pleomorphic RMS.

No reports to date have described the typical imaging findings and symptoms of RMS. Most reported cases were detected as a large mass of > 10 cm in diameter occupying a liver lobe. Our patient had a 12-cm liver mass, initially diagnosed and treated as a liver abscess, that caused peritoneal dissemination in line with the drainage route after resection. In the investigation of such cases, it is important to perform a percutaneous biopsy and include RMS as a differential diagnosis for liver masses in adults.

RMS in adults is a highly malignant tumor with a poor prognosis because of the absence of a standard treatment protocol. Sultan et al. [15] reported that RMS in adults had significantly poorer outcomes than in childhood (mean 5-year overall survival rates, 27% ± 1.4 and 61% ± 1.4%, respectively; *P* < 0.0001). Among previously reported cases of RMS originating in the liver, only two patients survived longer than 12 months; most patients died within 12 months from onset of the initial symptoms. It is necessary to establish the optimal treatment protocol and thus improve the outcome of patients with this rare but fatal cancer.

Radical resection with negative margins, chemotherapy, and radiotherapy are suggested by the Intergroup Rhabdomyosarcoma Study Group; these interventions constitute the generally optimal treatment protocol in childhood [16, 17]. Chemotherapeutic drugs include actinomycin D, vincristine, doxorubicin, cyclophosphamide, etoposide, and ifosfamide. We treated our patient’s RMS with trabectedin, as for other soft tissue sarcomas, because multi-drug combination therapy is considered difficult because of the worsening performance status. Our patient initially showed a good response to chemotherapy; however, she could not continue further chemotherapy because of severe adverse effects.

We have herein reported an extremely rare case of pleomorphic RMS of the liver in an adult. The rarity of this case is due to the location of the tumor and the age of the patient, and its reporting will help to establish standard diagnosis and treatment.

## Data Availability

The datasets used and/or analyzed during the current study are available from the corresponding author on reasonable request.

## Abbreviations

RMS: Rhabdomyosarcoma
CT: Computed tomography
